# 2,2′-[Octane-1,8-diyldioxy­bis(nitrilo­methyl­idyne)]diphenol

**DOI:** 10.1107/S160053680905466X

**Published:** 2009-12-24

**Authors:** Xiao-Jun Li, Jun-Feng Tong, Shang-Sheng Gong, Jian-Chao Wu, Li Xu

**Affiliations:** a Zhaosheng Office of Gansu Province, Lanzhou 730030, People’s Republic of China; b School of Chemical and Biological Engineering, Lanzhou Jiaotong University, Lanzhou 730070, People’s Republic of China

## Abstract

The complete mol­ecule of the title compound, C_22_H_28_N_2_O_4_, is generated by a crystallographic inversion centre at the mid-point of the central C—C bond. The two benzene rings are parallel to each other with a perpendicular inter­planar spacing of 1.488 (2) Å. Intra­molecular O—H⋯N hydrogen bonds generate two six-membered rings with *S*(6) motifs. In the crystal, weak inter­molecular C—H⋯O hydrogen bonds link neighbouring mol­ecules into an infinite three-dimensional network, which is further stabilized by weak C—H⋯π inter­actions.

## Related literature

For background to oxime-based salen-type tetra­dentate ligands, see: Dong *et al.* (2007 ▶, 2008 ▶); Dong, He *et al.* (2009 ▶); Kanderal *et al.* (2005 ▶); Fritsky *et al.* (2006 ▶). For the synthesis, see: Dong, Zhao *et al.* (2009 ▶).
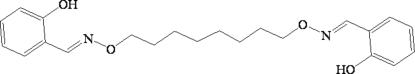


## Experimental

### 

#### Crystal data

C_22_H_28_N_2_O_4_
*M* *_r_* = 384.46
Monoclinic, 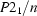

*a* = 10.5003 (12) Å
*b* = 5.3607 (8) Å
*c* = 18.612 (2) Å
β = 92.909 (1)°
*V* = 1046.3 (2) Å^3^
*Z* = 2
Mo *K*α radiation
μ = 0.08 mm^−1^
*T* = 293 K
0.32 × 0.16 × 0.07 mm

#### Data collection

Bruker SMART 1000 CCD area-detector diffractometer
Absorption correction: multi-scan (*SADABS*; Sheldrick, 1996 ▶) *T*_min_ = 0.974, *T*_max_ = 0.994
5181 measured reflections
1849 independent reflections
1109 reflections with *I* > 2σ(*I*)
*R*_int_ = 0.047

#### Refinement

*R*[*F* ^2^ > 2σ(*F* ^2^)] = 0.044
*wR*(*F* ^2^) = 0.110
*S* = 1.02
1849 reflections
127 parameters
H-atom parameters constrained
Δρ_max_ = 0.12 e Å^−3^
Δρ_min_ = −0.13 e Å^−3^

### 

Data collection: *SMART* (Siemens, 1996 ▶); cell refinement: *SAINT* (Siemens, 1996 ▶); data reduction: *SAINT*; program(s) used to solve structure: *SHELXS97* (Sheldrick, 2008 ▶); program(s) used to refine structure: *SHELXL97* (Sheldrick, 2008 ▶); molecular graphics: *SHELXTL* (Sheldrick, 2008 ▶); software used to prepare material for publication: *SHELXTL*.

## Supplementary Material

Crystal structure: contains datablocks global, I. DOI: 10.1107/S160053680905466X/hg2620sup1.cif

Structure factors: contains datablocks I. DOI: 10.1107/S160053680905466X/hg2620Isup2.hkl

Additional supplementary materials:  crystallographic information; 3D view; checkCIF report

## Figures and Tables

**Table 1:** Hydrogen-bond geometry (Å, °)

*D*—H⋯*A*	*D*—H	H⋯*A*	*D*⋯*A*	*D*—H⋯*A*
C11—H11⋯O1^i^	0.93	2.63	3.511 (2)	158
O2—H2⋯N1	0.82	1.92	2.634 (3)	145
C9—H9⋯*Cg*1	0.93	3.13	3.844 (2)	135

Symmetry code: (i) 
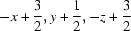
. *Cg*1 is the centroid of the C6–C11 ring.

**Fig. 1.**
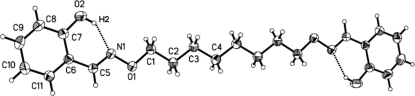
The molecule structure of the title compound with the atom numbering scheme [Symmetry codes: -x + 2,-y - 1,-z + 1]. Displacement ellipsoids for non-hydrogen atoms are drawn at the 30% probability level.

**Fig. 2.**
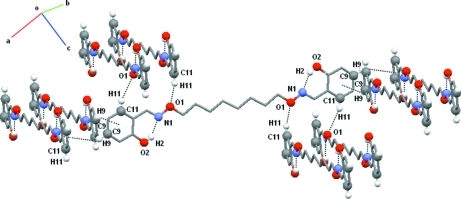
Part of supramolecular structure of the title compound along the b axis, formed by intra- and inter-molecule hydrogen bonds as well as C—H···π(Ph) interaction (dashed lines). H atoms not involved in hydrogen bonding have been omitted for clarity. Colour code: dark gray: C; red: O; pale blue: N.

**Fig. 3.**
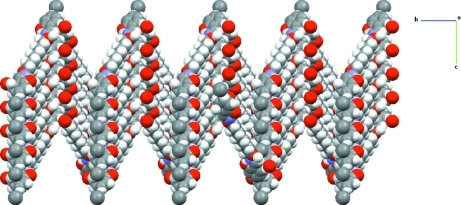
Part of zigzag supramolecular chains viewed along the b axis. Colour code: dark gray: C; red: O; pale blue: N; white: H.

## supplementary crystallographic information

### Comment

Much attention has been focused on oxime-based salen-type tetradentate ligands in recent years due to their high stability against imine metathesis reactions (Dong *et al.*, 2007; Dong *et al.* 2008; Dong, He *et al.* 2009). Also, the oxime ligands are strong donors and therefore the oxime-containing ligands were found to efficiently stabilize high oxidation states of metal ions like Cu(III) and Ni(III) (Kanderal *et al.*, 2005; Fritsky *et al.*, 2006). Herein, we report synthesis and structure of salen-type bis-oxime ligands, 2,2'-[1,1'-(octane-1,8-diyldioxydinitrilo)dimthylidyne]diphenol.

The centrosymmetric unit of the title compound (Fig. 1) is generated by a crystallographic inversion centre (symmetry code: -*x*, -*y*, -*z*) at the mid-point of the the central C—C bond and there is a crystallographic twofold screw axis (symmetry code: 1/2 - *x*, 1/2 + *y*, 1/2 - *z*). The two benzene rings of the title compound are parallel to each other with a perpendicular interplanar spacing of *ca* 1.488 (2) Å. In each title compound molecule, there exist two strong intramolecular O—H···N hydrogen bonds which generate two six-membered rings, producing two S(6) ring motifs (Fig. 1).

In the crystal structure, weak intermolecular C—H···O hydrogen bonds link neighbouring molecules into an infinite three-dimensional supramolecular structure (Table 1, Fig. 2) in which they may be effective in the stabilization of the structure. In additon, the crystal structure is further stabilized by C—H···π(Ph) interactions (C···π(centroid)= 3.844 (2) Å) (Fig. 2). With the help of intermolecular C—H···O hydrogen bonds and C—H···π(Ph) interactions, molecules form an infinite zigzag chain supramolecular frame viewed along *b* axis (Fig. 3).

### Experimental

2,2'-[1,1'-(Octane-1,8-diyldioxydinitrilo)dimethylidyne]diphenol was synthesized according to our previous work (Dong, Zhao *et al.*, 2009). To an ethanol solution (3 ml) of salicylaldehyde (326.1 mg, 2.67 mmol) was added an ethanol absolute (3 ml) of 1, 8-bis(aminooxy)octane (199.8 mg, 1.23 mmol). The mixture solution was stirred at 328–333 K for 24 h. After reaction solution was cooled to room temperature and Put aside for ten minutes, the white precipitate was formed. Then filtered under reduced pressure and washed successively with ethanol (2 ml) and n-hexane (6 ml), respectively. The product was dried under vacuum and purified with recrystallization from ethanol to yield 191.4 mg of the title compound. Yield, 49.93%. m. p. 348–349 K. Anal. Calcd. for C_22_H_28_N_2_O_4_: C, 68.73; H, 7.34; N, 7.29. Found: C, 68.65; H, 7.31; N, 7.41.

Colorless needle-like single crystals suitable for X-ray diffraction studies were obtained after one week by slow evaporation from a n-hexane solution of the title compound.

### Refinement

H atoms were placed in calculated positions and non-H atoms were refined anisotropically. The remaining H atoms were treated as riding atoms with distances C—H = 0.97 Å (CH_2_), 0.93 Å (CH), 0.82 Å (OH), and *U*_iso_(H) = 1.20 *U*_eq_(C), 1.50 *U*_eq_(O).

### Crystal data

**Table:**

C_22_H_28_N_2_O_4_	*F*(000) = 412
*M**_r_* = 384.46	*D*_x_ = 1.220 Mg m^−^^3^
Monoclinic, *P*2_1_/*n*	Melting point = 348–349 K
Hall symbol: -P 2yn	Mo *K*α radiation, λ = 0.71073 Å
*a* = 10.5003 (12) Å	Cell parameters from 1194 reflections
*b* = 5.3607 (8) Å	θ = 3.7–25.1°
*c* = 18.612 (2) Å	µ = 0.08 mm^−^^1^
β = 92.909 (1)°	*T* = 293 K
*V* = 1046.3 (2) Å^3^	Needle-like, colorless
*Z* = 2	0.32 × 0.16 × 0.07 mm

### Data collection

**Table:**

Bruker SMART 1000 CCD area-detector diffractometer	1849 independent reflections
Radiation source: fine-focus sealed tube	1109 reflections with *I* > 2σ(*I*)
graphite	*R*_int_ = 0.047
phi and ω scans	θ_max_ = 25.0°, θ_min_ = 2.2°
Absorption correction: multi-scan (*SADABS*; Sheldrick, 1996)	*h* = −12→12
*T*_min_ = 0.974, *T*_max_ = 0.994	*k* = −6→5
5181 measured reflections	*l* = −22→20

### Refinement

**Table:**

Refinement on *F*^2^	Primary atom site location: structure-invariant direct methods
Least-squares matrix: full	Secondary atom site location: difference Fourier map
*R*[*F*^2^ > 2σ(*F*^2^)] = 0.044	Hydrogen site location: inferred from neighbouring sites
*wR*(*F*^2^) = 0.110	H-atom parameters constrained
*S* = 1.02	*w* = 1/[σ^2^(*F*_o_^2^) + (0.0455*P*)^2^] where *P* = (*F*_o_^2^ + 2*F*_c_^2^)/3
1849 reflections	(Δ/σ)_max_ < 0.001
127 parameters	Δρ_max_ = 0.12 e Å^−^^3^
0 restraints	Δρ_min_ = −0.13 e Å^−^^3^

### Special details

**Table:**

Geometry. All e.s.d.'s (except the e.s.d. in the dihedral angle between two l.s. planes) are estimated using the full covariance matrix. The cell e.s.d.'s are taken into account individually in the estimation of e.s.d.'s in distances, angles and torsion angles; correlations between e.s.d.'s in cell parameters are only used when they are defined by crystal symmetry. An approximate (isotropic) treatment of cell e.s.d.'s is used for estimating e.s.d.'s involving l.s. planes.
Refinement. Refinement of *F*^2^ against ALL reflections. The weighted *R*-factor *wR* and goodness of fit *S* are based on *F*^2^, conventional *R*-factors *R* are based on *F*, with *F* set to zero for negative *F*^2^. The threshold expression of *F*^2^ > σ(*F*^2^) is used only for calculating *R*-factors(gt) *etc*. and is not relevant to the choice of reflections for refinement. *R*-factors based on *F*^2^ are statistically about twice as large as those based on *F*, and *R*- factors based on ALL data will be even larger.

### Fractional atomic coordinates and isotropic or equivalent isotropic displacement parameters (Å^2^)

**Table:**

	*x*	*y*	*z*	*U*_iso_*/*U*_eq_	
N1	0.62173 (12)	0.3737 (3)	0.63398 (8)	0.0525 (4)	
O1	0.72297 (10)	0.2054 (2)	0.64425 (6)	0.0594 (4)	
O2	0.42211 (13)	0.5954 (3)	0.57083 (7)	0.0901 (5)	
H2	0.4800	0.4929	0.5744	0.135*	
C1	0.73413 (16)	0.0613 (4)	0.58033 (9)	0.0550 (5)	
H1A	0.7458	0.1706	0.5396	0.066*	
H1B	0.6573	−0.0359	0.5706	0.066*	
C2	0.84680 (16)	−0.1083 (3)	0.59128 (9)	0.0531 (5)	
H2A	0.8319	−0.2220	0.6305	0.064*	
H2B	0.9216	−0.0095	0.6048	0.064*	
C3	0.87221 (16)	−0.2569 (3)	0.52490 (9)	0.0544 (5)	
H3A	0.8860	−0.1424	0.4857	0.065*	
H3B	0.7972	−0.3554	0.5116	0.065*	
C4	0.98565 (16)	−0.4291 (3)	0.53369 (8)	0.0533 (5)	
H4A	0.9704	−0.5478	0.5716	0.064*	
H4B	1.0600	−0.3315	0.5488	0.064*	
C5	0.60867 (15)	0.5122 (3)	0.68811 (9)	0.0500 (5)	
H5	0.6636	0.4919	0.7285	0.060*	
C6	0.51094 (14)	0.7007 (3)	0.68884 (9)	0.0467 (5)	
C7	0.42313 (17)	0.7368 (4)	0.63135 (10)	0.0604 (5)	
C8	0.33222 (18)	0.9214 (4)	0.63483 (12)	0.0762 (6)	
H8	0.2727	0.9431	0.5966	0.091*	
C9	0.32837 (18)	1.0723 (4)	0.69355 (12)	0.0719 (6)	
H9	0.2671	1.1972	0.6948	0.086*	
C10	0.41430 (17)	1.0414 (4)	0.75083 (11)	0.0636 (6)	
H10	0.4116	1.1438	0.7911	0.076*	
C11	0.50416 (16)	0.8572 (3)	0.74777 (10)	0.0561 (5)	
H11	0.5626	0.8365	0.7865	0.067*	

### Atomic displacement parameters (Å^2^)

**Table:**

	*U*^11^	*U*^22^	*U*^33^	*U*^12^	*U*^13^	*U*^23^
N1	0.0484 (8)	0.0534 (10)	0.0557 (10)	0.0098 (8)	0.0005 (7)	−0.0013 (8)
O1	0.0566 (7)	0.0647 (9)	0.0562 (8)	0.0184 (7)	−0.0025 (6)	−0.0073 (7)
O2	0.0960 (10)	0.1020 (12)	0.0687 (10)	0.0358 (10)	−0.0303 (8)	−0.0248 (9)
C1	0.0586 (11)	0.0525 (12)	0.0542 (12)	0.0051 (10)	0.0037 (9)	−0.0047 (9)
C2	0.0550 (10)	0.0472 (11)	0.0574 (12)	0.0033 (10)	0.0049 (8)	−0.0006 (9)
C3	0.0582 (11)	0.0475 (12)	0.0574 (11)	0.0047 (10)	0.0018 (9)	−0.0020 (10)
C4	0.0598 (10)	0.0450 (11)	0.0549 (11)	0.0050 (9)	0.0018 (9)	0.0017 (9)
C5	0.0478 (10)	0.0566 (12)	0.0454 (11)	0.0038 (9)	0.0012 (8)	0.0004 (10)
C6	0.0447 (10)	0.0476 (11)	0.0477 (11)	0.0005 (9)	0.0027 (8)	0.0030 (9)
C7	0.0610 (11)	0.0635 (14)	0.0560 (12)	0.0096 (11)	−0.0055 (9)	−0.0046 (11)
C8	0.0681 (13)	0.0830 (17)	0.0756 (15)	0.0231 (13)	−0.0155 (11)	−0.0012 (13)
C9	0.0627 (12)	0.0621 (14)	0.0912 (17)	0.0176 (11)	0.0062 (12)	0.0015 (13)
C10	0.0648 (12)	0.0577 (14)	0.0693 (14)	0.0042 (11)	0.0129 (10)	−0.0093 (11)
C11	0.0538 (10)	0.0603 (13)	0.0542 (12)	0.0001 (10)	0.0026 (9)	−0.0028 (10)

### Geometric parameters (Å, °)

**Table:**

N1—C5	1.265 (2)	C4—H4A	0.9700
N1—O1	1.3997 (16)	C4—H4B	0.9700
O1—C1	1.4281 (19)	C5—C6	1.441 (2)
O2—C7	1.357 (2)	C5—H5	0.9300
O2—H2	0.8200	C6—C11	1.386 (2)
C1—C2	1.498 (2)	C6—C7	1.390 (2)
C1—H1A	0.9700	C7—C8	1.379 (3)
C1—H1B	0.9700	C8—C9	1.362 (3)
C2—C3	1.505 (2)	C8—H8	0.9300
C2—H2A	0.9700	C9—C10	1.371 (2)
C2—H2B	0.9700	C9—H9	0.9300
C3—C4	1.509 (2)	C10—C11	1.369 (2)
C3—H3A	0.9700	C10—H10	0.9300
C3—H3B	0.9700	C11—H11	0.9300
C4—C4^i^	1.510 (3)		
			
C5—N1—O1	112.46 (14)	C3—C4—H4B	108.7
N1—O1—C1	109.18 (12)	C4^i^—C4—H4B	108.7
C7—O2—H2	109.5	H4A—C4—H4B	107.6
O1—C1—C2	108.22 (14)	N1—C5—C6	121.70 (15)
O1—C1—H1A	110.1	N1—C5—H5	119.1
C2—C1—H1A	110.1	C6—C5—H5	119.1
O1—C1—H1B	110.1	C11—C6—C7	117.79 (16)
C2—C1—H1B	110.1	C11—C6—C5	119.83 (15)
H1A—C1—H1B	108.4	C7—C6—C5	122.37 (16)
C1—C2—C3	112.42 (15)	O2—C7—C8	117.61 (17)
C1—C2—H2A	109.1	O2—C7—C6	122.55 (17)
C3—C2—H2A	109.1	C8—C7—C6	119.84 (18)
C1—C2—H2B	109.1	C9—C8—C7	120.86 (18)
C3—C2—H2B	109.1	C9—C8—H8	119.6
H2A—C2—H2B	107.9	C7—C8—H8	119.6
C2—C3—C4	113.99 (14)	C8—C9—C10	120.44 (19)
C2—C3—H3A	108.8	C8—C9—H9	119.8
C4—C3—H3A	108.8	C10—C9—H9	119.8
C2—C3—H3B	108.8	C11—C10—C9	118.86 (19)
C4—C3—H3B	108.8	C11—C10—H10	120.6
H3A—C3—H3B	107.7	C9—C10—H10	120.6
C3—C4—C4^i^	114.06 (17)	C10—C11—C6	122.20 (17)
C3—C4—H4A	108.7	C10—C11—H11	118.9
C4^i^—C4—H4A	108.7	C6—C11—H11	118.9
			
C5—N1—O1—C1	−178.24 (15)	C11—C6—C7—C8	−0.9 (3)
N1—O1—C1—C2	177.28 (13)	C5—C6—C7—C8	−179.52 (17)
O1—C1—C2—C3	−176.21 (14)	O2—C7—C8—C9	−179.25 (19)
C1—C2—C3—C4	179.63 (15)	C6—C7—C8—C9	1.1 (3)
C2—C3—C4—C4^i^	−177.76 (19)	C7—C8—C9—C10	−0.8 (3)
O1—N1—C5—C6	179.40 (14)	C8—C9—C10—C11	0.3 (3)
N1—C5—C6—C11	−176.61 (17)	C9—C10—C11—C6	−0.2 (3)
N1—C5—C6—C7	1.9 (3)	C7—C6—C11—C10	0.5 (3)
C11—C6—C7—O2	179.42 (17)	C5—C6—C11—C10	179.12 (16)
C5—C6—C7—O2	0.8 (3)		

Symmetry codes: (i) −*x*+2, −*y*−1, −*z*+1.

### Hydrogen-bond geometry (Å, °)

**Table:**

Cg1 is the centroid of the C6–C11 ring.

**Table:**

*D*—H···*A*	*D*—H	H···*A*	*D*···*A*	*D*—H···*A*
C11—H11···O1^ii^	0.93	2.63	3.511 (2)	158
O2—H2···N1	0.82	1.92	2.634 (3)	145
C9—H9···Cg1	0.93	3.13	3.844 (2)	135

Symmetry codes: (ii) −*x*+3/2, *y*+1/2, −*z*+3/2.
